# Callus Derived from Petals of the *Rosa hybrida* Breeding Line 15R-12-2 as New Material Useful for Fragrance Production

**DOI:** 10.3390/plants12162986

**Published:** 2023-08-18

**Authors:** Ka Youn Lee, Ju Young Shin, Myung Suk Ahn, Se Jin Kim, Hye Ryun An, Yae Jin Kim, O Hyeon Kwon, Su Young Lee

**Affiliations:** Floriculture Research Division, National Institute of Horticultural and Herbal Science, Rural Development Administration (RDA), Wanju 55365, Republic of Korea; kayoun200@korea.kr (K.Y.L.); juyoung72@korea.kr (J.Y.S.); ahnms@korea.kr (M.S.A.); sejin0905@korea.kr (S.J.K.); hryun@korea.kr (H.R.A.); yj0503@korea.kr (Y.J.K.); rkddnjseo01@korea.kr (O.H.K.)

**Keywords:** rose (*Rosa hybrida*), callus induction, volatile organic compounds, headspace solid-phase microextraction coupled to chromatography–mass spectrometry, VOC synthesis gene expression

## Abstract

Rose (*Rosa hybrida*) is a major flower crop worldwide and has long been loved for its variety of colors and scents. Roses are mainly used for gardening or cutting flowers and are also used as raw materials for perfumes, cosmetics, and food. Essential oils, which are extracted from the flowers of plants, including roses, have various scents, and the essential oil market has been growing steadily owing to the growing awareness of the benefits of natural and organic products. Therefore, it is necessary to develop a system that stably supplies raw materials with uniform ingredients in line with the continuous increase in demand. In this study, conditions for the efficient induction of callus were established from the petals of the rose breeding line 15R-12-2, which has a strong scent developed by the National Institute of Horticultural and Herbal Science, Rural Development Administration. The highest callus induction rate (65%) was observed when the petals of the fully open flower (FOF) were placed on the SH11DP medium so that the abaxial surface was in contact with the medium. In addition, the VOCs contained in the petals of 15R-12-2 and the petal-derived callus were analyzed by HS-SPME-GC-MS. Thirty components, including esters and alcohols, were detected in the petal-derived callus. Among them, 2-ethylhexan-1-ol, which showed 59.01% relative content when extracted with hexane as a solvent, was the same component as detected in petals. Therefore, petal-derived callus is expected to be of high industrial value and can be suggested as an alternative pathway to obtaining VOCs.

## 1. Introduction

Rose (*Rosa hybrida*) is a major flower crop worldwide and has long been loved for its variety of colors and scents. Roses are mainly used for gardening or cutting flowers and are also used as raw materials for perfumes, cosmetics, and food. Essential oils extracted from the flowers of plants, including roses, have various scents depending on the chemical group and composition of volatile organic compounds (VOCs) [1]. Most of the components of floral scents are divided into terpenes (including monoterpenes and sesquiterpenes), phenylpropanoids, and fatty acid derivatives [2,3]. The fragrance of roses comprises more than 400 VOCs, including phenylpropanoids, fatty acid derivatives, alcohols, aldehydes, esters, and terpenes. Esters are typical rose scents and include geranyl acetate and 2-phenylethyl acetate [4]. These VOCs are emitted from the aerial parts, such as petals, sepals, and stamens. Bergougnoux et al. [5] reported that the sepals of rose produce five times lower fatty acid derivatives (per 1 g fresh weight) than the petals and stamens. By contrast, high levels of monoterpene alcohol were released from the petals and stamens, and the concentrations per gram of fresh weight were similar. However, the petals are still used as the main material for oil production, as they account for more than 90% of the fresh weight of flowers. Recently, the essential oil market has been growing steadily owing to the growing awareness of the benefits of natural and organic products. The global essential oils market size reached USD 8.74 billion in 2020 and is expected to grow at a CAGR of 9.57% from 2021 to 2028 [6]. Rose essential oil contains large amounts of VOCs; therefore, it is used in aromatherapy, food and beverages, pharmaceuticals, and perfumes. However, rose oil produced in Turkey is traded at a very high price because its content is low enough to obtain only 1 kg of oil from approximately 3000 kg of *Rosa damascene* petals [7]. In addition, because essential oil is extracted from fresh flowers harvested from late May to late June, it is affected by various factors such as temperature, humidity, and precipitation. Therefore, it is necessary to develop a system that stably supplies raw materials with uniform ingredients in line with the continuous increase in demand [8].

Raw materials produced through plant cell cultures are free from pesticides and are independent of climate because they are produced under optimized and controlled conditions [9]. A representative compound produced through cell culture is anthocyanin, which has been studied in various plants such as *Aralia cordata*, *Vitis vinifera*, and *Daucus carota* L. [10,11,12]. In addition, studies are being conducted to identify the major active ingredients of medicinal plants, and the ginsenosides of ginseng are representative of these [13,14,15]. Furthermore, studies are being conducted on the application of this extract in cosmetics. Park et al. [16] confirmed that the main components of the callus extract of *Pyrus pyrifolia* are uridine (1), adenosine (2), and guanosine (3), and reported that the wound recovery rates of keratinocytes and fibroblasts increased after treatment with the extract.

Previously, the Rural Development Administration’s National Institute of Horticultural and Herbal Science developed 15R-12-2, a rose breeding line with a strong fragrance. In this study, conditions for the efficient induction of callus were established from the petals of the rose breeding line 15R-12-2. In addition, the VOCs of the 15R-12-2 petals and the petal-derived callus were analyzed by HS-SPME-GC-MS to evaluate their potential as new raw materials for fragrances.

## 2. Results and Discussion

### 2.1. Callus Induction

**Figure 1:**
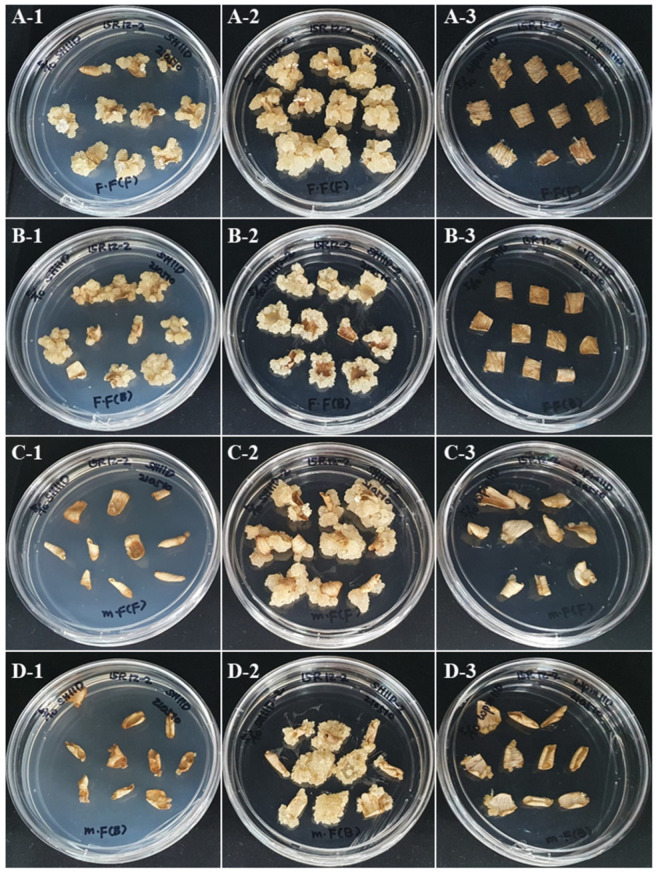
Appearance of callus induced from petal explants after 7 weeks of culture without light conditions. (**A**) The abaxial side of the FOF petal was placed in contact with the culture medium. (**B**) The adaxial side of the FOF petal was placed in contact with the culture medium. (**C**) The abaxial side of the POF petals is placed in contact with the culture medium. (**D**) The adaxial side of the POF petals is placed in contact with the culture medium. Numbers represent the names of each medium. 1: SH11D, 2: SH11DP, 3: WPM11D.

**Figure 2:**
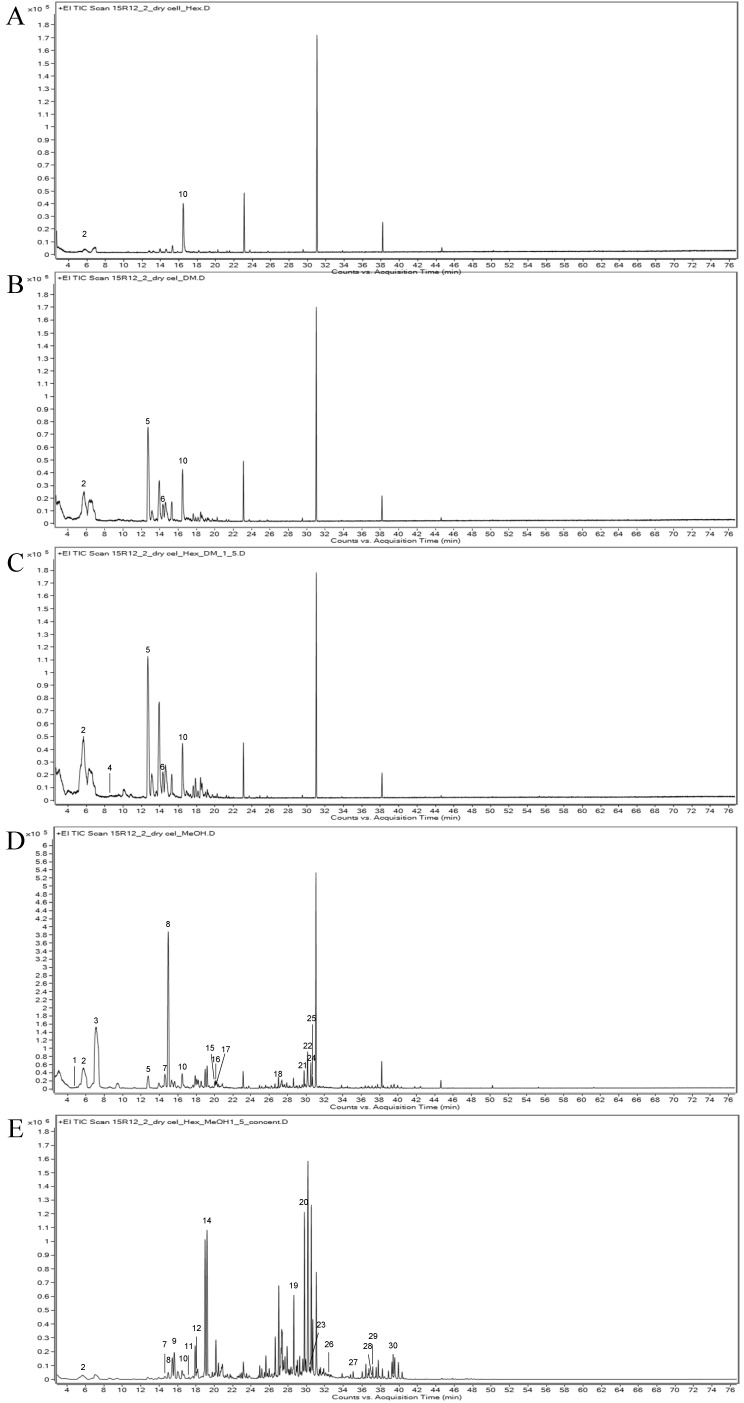
Component analysis of petal-derived callus extracts by gas chromatography–mass spectrometry. The extraction solvents used were hexane (**A**), dichloromethane (**B**), hexane:dichloromethane (1:5) (**C**), methanol (**D**), and hexane:methanol (1:5) (**E**).

As a result of examining the callus induction rate after culturing the petal explant in the dark for 4 weeks, the highest induction rate of 65% was observed when the petals of the FOF were placed on the SH11DP medium so that the abaxial surface was in contact with the medium (Figure 1A-2 and Table 1).

Callus formation was generally better in the fully open flower (FOF) than in the partially open flower (POF) stage, and the explant orientation did not have a significant effect on callus induction. Because callus formation is a process of dedifferentiation, immature explant tissue is more likely to induce callus formation than mature tissue. Therefore, we expected that the petals of POF would be more effective in inducing callus than the petals of FOF. However, the results of this study showed that the petals of FOF had a high callus induction rate and a low browning rate in SH11DP and SH11D media (Figure 1 and Table 1). The browning of explants during tissue culture is related to oxidation. Duan et al. [17] reported that when callus is induced from the leaves of *Isodon amethystoides*, the content of polyphenols is reduced and the activity of polyphenol oxidase (PPO), which promotes oxidation, is increased in aged leaves compared to young leaves, which affects the browning of explants. Zhou et al. [18] measured the content of flavonoids, phenols, and antioxidant substances in the flowers of *Eriobotrya japonica* Lindl. and confirmed that the content and antioxidant activity were high in the full-bloom stage. In addition, Fu et al. [19] measured the contents of catechin, chlorogenic acid, rutin, and quercetin in each flower stage of daylily flowers and found that the content of catechin and antioxidant capacity were the highest in the full-bloom stage. Catechin, an antioxidant, accounted for 74.11% of the phenolic compounds in daylily flowers. Schmizer et al. [20] reported that the content of six flavonols belonging to the quercetin group was higher in white roses than in colored roses and the content was highest in full bloom. Therefore, it is thought that the white rose breeding line 15R-12-2 used in this study was effective in suppressing browning and inducing callus formation because the antioxidant capacity of the petals of FOF was higher than that of POF.

In a comparison of the callus induction rates when the abaxial and adaxial surfaces were placed facing the culture medium, the callus induction rate was high when the abaxial surface was placed in contact with the culture medium (Figure 1 and Table 1). The leaves of most plants were hypostomatic, with a wax layer on the adaxial surface and numerous pores on the abaxial surface. By contrast, in the case of petals, the abaxial surface had a thicker cell wall and a well-developed cuticle compared to the adaxial surface. In addition, according to Sulborska et al. [21], in the case of flower petals, the adaxial surface has papillae, whereas the abaxial surface is composed of cells with flat outer walls and no pores. In the case of rose petals, it has been reported that the adaxial epidermis has many papillae, whereas the hypocotyl epidermis is composed of a flat and thick cell wall [5,22]. Two steps are required for callus formation: (1) degradation of the outer cell wall and (2) synthesis of new cells [23]. These results suggested that the adaxial side, which has a relatively thin cell wall, may be more advantageous for callus formation than the abaxial side. This supports our finding that callus induction is active on the adaxial side.

**Figure 3:**
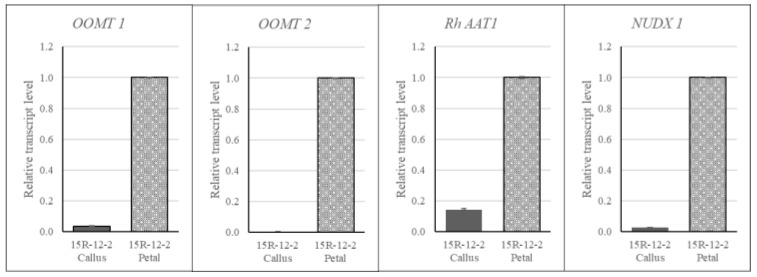
Comparative analysis of the relative expression of VOC biosynthetic genes in rose petals and petal-derived callus using qRT-PCR.

A comparison of callus induction rates according to medium composition showed that SH11DP was the most effective. The callus was slightly yellow, friable, had high water content, and grew rapidly (Figure 1A–D-2). By contrast, in the WPM11D medium, a small amount of callus was induced, but most of the sections were browned (Figure 1A–D-3). Beruto et al. [24] reported that the physicochemical characteristics of callus may vary depending on the type of gelling agent, which affects the differences in water and nutrients available to the explant. In this study, we found that the callus formation rate was higher in the SH11DP medium than in the SH11D medium. This is thought to be because phytagel absorbs water and nutrients more easily than agar. In addition, agar is composed of two polysaccharides, agarose and agaropectin, whereas phytagel is composed of only one water-soluble anionic polysaccharide, which is thought to cause a difference in the callus formation rate depending on its composition [25].

### 2.2. Analysis of the VOCs of Petal-Derived Callus in Rose

To identify the various VOCs in the callus of the 15R-12-2 line, extracts were prepared using five solvents and analyzed using HS-SPME-GC-MS. The peaks obtained by GC-MS analysis were compared with mass spectral library data, and a total of 30 peaks were identified and estimated (Figure 2 and Table 2). The number of detected components differed depending on the solvent; hexane:methanol (1:5) (16 species), methanol (15 species), hexane:dichloromethane (1:5) (6 species), dichloromethane (4 species), and hexane (2 species). Among these components, hexanal, butan-2-amine, 6-methylhept-5-en-2-one, 2-ethylhexan-1-ol, (Z)-oct-2-en-1-ol, and nonanal were identified as the components used for fragrances. Hexanal and 2-ethylhexan-1-ol were identified in all five solvents.

When comparing the VOCs of the petal and petal-derived callus extracts, 2-ethylhexan-1-ol was detected in both (Table 2 and Table S1). The content of 2-ethylhexan-1-ol, a sweet and refreshing fragrance compound found in roses, was the highest (59.01%) in petal-derived callus when hexane was used as a solvent. In addition, hexanal, which contributes to the flavor of fresh grass, showed high contents of 13.07% and 13.45% in the hexane and hexane:dichloromethane (1:5) solvents, respectively. 6-methylhept-5-en-2-one is a tangerine-like lemongrass flavor detected at 3.76% and 3.09% in dichloromethane and hexane:dichloromethane (1:5), respectively. In addition, small amounts of butan-2-amine (0.02%), (Z)-oct-2-en-1-ol (1.19%), and nonanal (0.30%) were detected in the hexane:dichloromethane (1:5) and methanol solvent extracts (Table 2).

Biomass production using callus is one of the methods widely used as a production platform because it enables a stable supply of raw materials without contamination. Moreover, cells grown in vitro have a much more diverse secondary metabolite profile than plants grown in soil, and the profile of secondary metabolites may be altered. Therefore, new and pure compounds can be synthesized through cell culture. Furthermore, studies have been conducted to increase functional substance content via direct or indirect stress induction and plastid biosynthesis. Ram et al. [26] reported that culture callus derived from the leaf segments of *R. hybrida* cv. Pusa Ajay in a medium containing 204.5 mM sucrose and 0.50 μM MeJA produced 1.56 ± 0.03 CV g^−1^ Fresh weight (FW), which increased anthocyanins compared to the control (1.15 ± 0.05 CV g^−1^ FW). In addition, Darwich and Ahmend [27] reported that the production of phenolic compounds from callus derived from *Rosa damascena* petals increased by 37.2–153.3% compared to the control through the addition of abiotic and biotic elicitors and jasmine oil.

Among the callus components detected in the present study, hexanal and 2-ethylhexan-1-ol have been used for industrial purposes in various fields since ancient times. Hexanal reduces fruit necrosis by suppressing the maturation and growth of pathogens such as bacteria and fungi [28]. It is also a safe ingredient used as a flavoring substance for producing fruit flavors in foods [29]. 2-ethylhexan-1-ol emits a soft and sweet rose scent and is used as a raw material for sunscreens and anti-aging cosmetics [30]. In addition, they are widely used in non-cosmetic products such as shampoos, soaps, and air fresheners [31]. These reports suggest that the rose-petal-derived callus used in this study is a promising material for the production of functional substances.

### 2.3. Expression Analysis of Genes Involved in the Biosynthesis of VOCs of Rose-Petal-Derived Callus

To compare the expression levels of genes involved in the biosynthesis of VOCs, RNA was extracted from petals and petal-derived callus of rose, and qRT-PCR was performed targeting *OOMT1*, *OOMT2*, *RhAAT1*, and *NUDX1*. As a result, it was found that the expression level was lower in the callus than in the petals as a whole, and among them, the expression level of *RhAAT1* was relatively higher than that of other genes in petal-derived callus (Figure 3).

Esters are produced by the enzyme alcohol acetyltransferase (AAT), which catalyzes the reaction between various alcohols and Acetyl-CoA [32,33]. They are present in flowers and fruits and are used in small amounts in beverages, cosmetics, and fragrances. However, they are widely used because they play important roles in taste and aroma [34,35,36]. Owing to the substrate specificity of the AAT enzyme, the esters produced were expressed differently. Shalit et al. [4] measured the levels of volatile acetate esters by dividing the flower development stages of the rose cultivar Fragrant Cloud and identified the major volatile esters. The major esters were 2-phenylethyl acetate, cis-3-hexenyl acetate, geranyl acetate, and citronellyl acetate, which showed the highest concentrations in full bloom. In addition, we confirmed that the expression level of *RhAAT*, a gene encoding the AAT enzyme in roses, increased as flowering progressed. Zhu et al. [37] reported that the release of alcohol and esters, and the expression of *AAT1* increased as fruit ripening progressed in two apple cultivars. In particular, this confirms that the main esters of the two varieties are different, and the main volatile ester of ‘Granny Smith’ is hexanal. In this study, esters such as hexanal (13.45% based on hexane:dichloromethane (1:5) solvent extraction) and dimethyl oxalate (22.83% based on methanol solvent extraction) were detected in the callus of 15R-12-2. It is thought that the action of the AAT enzyme may have occurred because a large amount of this component was detected. In addition, the result showing a high expression level (10%) of the *RhAAT1* gene compared to that in the petals supports this interpretation.

The biosynthesis of monoterpenes, which account for a large proportion of the fragrance-forming components in flowers, is carried out by plastid-localized terpene synthases (TPSs), including geraniol synthase (GES) [38,39,40,41]. However, in the case of roses, the cytoplasmic Nudix hydrolase RhNUDX1 is involved in monoterpene alcohol biosynthesis. Among these, geraniol is produced by RhNUDX1, which includes diphosphohydrolase that converts geranyl diphosphate to monophosphate and is hydrolyzed by phosphatase activity [42]. Geraniol produced in this manner imparts a sweet fragrance to roses. We analyzed the VOCs in the petals of the rose breeding line 15R-12-2, and monoterpenes or monoterpenoids, including (E)-methyl geranate, beta-myrcene, and citral, were detected (Table S1 and Figure S1). However, no components of the monoterpene group were detected in the callus of 15R-12-2 used in this study. Based on a comparison of *RhNUDX1* gene expression levels, it was considered that it was biosynthesized in a very small amount within the callus, as it was very low in the callus compared to the petal.

Finally, DMT is derived from orcinol and biosynthesized through the last two steps of methylation. Orcinol is methylated to 3-methoxy-5-hydroxy toluene by orcinol O-methyl transferase 1 (*OOMT1*) and then to *OOMT2* to form the final DMT [43]. OOMT also catalyzes the second and third methylation reactions in the biosynthetic pathway of 1,3,5-trimethoxybenzene (TMB) derived from phloroglucinol [44,45]. DMT and TMB were detected at 17.73% and 0.02%, respectively, in the petals of the rose line 15R-12-2 but were not detected in the callus. In addition, the expression levels of two genes (*OOMT1* and *OOMT2*) involved in biosynthesis were also confirmed to be very low in the callus compared with those in the petals. These results suggest that OOMT accumulation and its catalytic reaction were not performed smoothly in the callus, which is a mass of dedifferentiated cells compared to the petal tissue; OOMT is mainly confined to the cone-shaped cells of the petal and accumulated tissue [46]. However, because the expression level of *OOMT1* was approximately 8% higher than that of *OOMT2*, it was expected that a small amount of the intermediate product was produced because of the catalyst of *OOMT1*.

## 3. Materials and Method

### 3.1. Derivation of Callus from Petals

#### 3.1.1. Explant Material and Surface Sterilization

**Figure 4:**
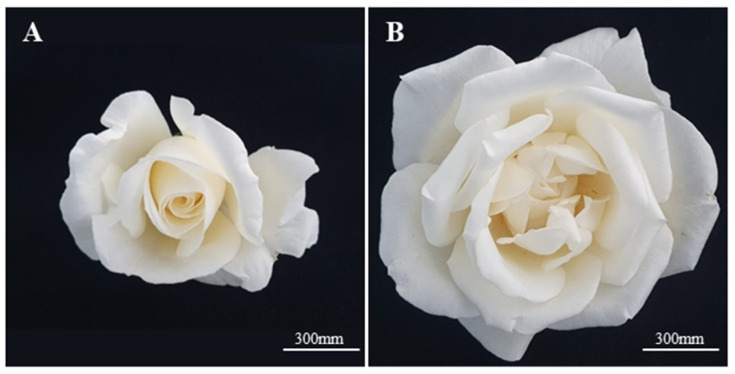
The flowers of the rose breeding line 15R-12-2 were used in this study. (**A**) Partially open flower (POF); (**B**) fully open flower (FOF).

The rose breeding line 15R-12-2 used in the experiment was rooted on perlite (Kyung dong one, Seoul, Republic of Korea), irrigated with Hoagland nutrient solution (manufactured and used according to the manufacturer’s instructions) [47], and grown in a glasshouse at the National Institute of Horticultural and Herbal Science under controlled conditions, with an average temperature of 25 ± 2 °C and 50 ± 5 humidity. Flowers were collected at both stages (POF, FOF) to identify the flowering stage suitable for callus induction (Figure 4). Petals were first washed under running water for 10 min, surface-sterilized with 70% EtOH for 15 s, washed three times with sterile water, and immersed in 0.5% NaOCl for 30 s to 3 min, according to the condition of the petals. The sterilized petals were washed several times with sterile water and cut into 0.5 × 0.5 cm^2^ explants; the explants were placed with the adaxial side and abaxial side in contact with the medium and cultured without light at 25 ± 1 °C for 7 weeks.

#### 3.1.2. Condition of the Medium and Culture Used to Induce Callus from the Petals

As for the medium, 30 g·L^−1^ of sucrose (Duchefa, Haarlem, The Netherlands) and 11 mg·L^−1^ of 2, 4-dichlorophenoxyacetic acid (2, 4-D, Duchefa, Haarlem, The Netherlands) were added to the basic Schenk and Hildebrandt medium (SH, Duchefa, Haarlem, The Netherlands) and woody plant medium (WPM, Duchefa, Haarlem, The Netherlands) [48,49]. The media were solidified with plant agar (Duchefa, Haarlem, The Netherlands) and Phytagel (Duchefa, Haarlem, The Netherlands), respectively, and the pH was adjusted to 5.8. The meanings of the abbreviations for the media are as follows: SH11D, medium solidified with plant agar 7.2 g·L^−1^ to SH basal media; SH11DP, medium solidified with phytagel 2.4 g·L^−1^ to SH basal media; WPM11D, medium solidified with plant agar 7.2 g·L^−1^ to WPM basal media. The prepared media were sterilized by autoclaving at 121 °C for 20 min, and then 30 mL of each was dispensed into a Petri dish (100 mm × 20 mm). The explants were placed in 10 pieces per dish and cultured in the dark at 25 °C ± 1 °C for 7 weeks.

### 3.2. Extract and Identification of VOC Analysis of Petal-Derived Callus Using HS-SPME/GC-MS

HS-SPME-GC-MS was performed to analyze the VOCs in the petals of 15R-12-2 and petal-derived callus. Fresh callus cultured for 4 weeks in SH11DP medium, which had the highest callus formation rate, and petals grown in a greenhouse were weighed after harvest, immediately frozen in liquid nitrogen, and stored at −80 °C. Prior to the experiment, the samples were dried by lyophilization (LYCF-12015; Operon, Republic of Korea) and used after being powdered. To detect various VOCs, callus extracts were prepared using a total of 5 solvents: hexane, dichloromethane, hexane:dichloromethane (1:5), methanol, and hexane:methanol (1:5). After adding 3 mL of solvent to 0.1 g of powdered sample, the VOCs were extracted by vortexing for 5 min and allowing to stand for 3 h. The supernatant collected by centrifugation (13,000 rpm for 10 min) was filtered with a 0.2 μm filter and dried using a solvent lyophilizer (FD5518S, Ilshin, Republic of Korea). The extract was placed in a 20 mL vial and sealed. The solution was equilibrating while being stirred at 40 °C for 10 min before injection, an SPME fiber needle was inserted, and VOCs were extracted for 30 min. After sampling, SPME was inserted into a GC-MS injector at 230 degrees and desorbed.

The VOCs were obtained using HS-SPME and analyzed after injection into a GC-MS system (Agilent, 7890B GC system, 5977B MSD, Santa Clara, CA, USA). The column used was an HP-5MS capillary column (Agilent, Santa Clara, CA, USA), and the mobile phase gas was helium at a flow rate of 1 mL/min. The oven was maintained at 40 °C for 5 min, then raised to 240 °C at 3 °C per minute and maintained for 5 min. The analysis mode was spotless, and the temperature of the inlet was set to 230 °C. A mass spectrometer was used for full-scan mode scanning at m/z 40–500, and the mass spectrum was measured at 70 eV. To identify directional components, the National Institute of Standards and Technology (NIST) 14 library was used, and their spectral matches were confirmed using MassHunter Qualitative Analysis Workflows software version B. 08.00.

### 3.3. Analysis of Gene Expression Involved in the Biosynthesis of VOCs Using Quantitative Real-Time Polymerase Chain Reaction (qRT-PCR)

qRT-PCR was performed to analyze the expression of genes involved in the VOC biosynthesis of the callus. Callus cultured in SH11DP medium and petals of 15R-12-2 grown in a glasshouse were collected, rapidly frozen in liquid nitrogen, and then stored at −80 °C. Samples were powdered using a mortar and pestle, and polysaccharides and polyphenols were removed using Fruit-mate™ for RNA purification (Takara, Japan) before RNA extraction. RNA was isolated from petals and petal-derived callus, respectively, according to the RNAiso Plus (Takara, Japan) manufacturer’s instructions, and cDNA (50 ng/μl) was synthesized using the PrimeScript™ RT reagent Kit with gDNA Eraser (Takara, Japan). qRT-PCR was performed with 2 ul cDNA using CFX Opus 96 (Bio-Rad, Hercules, CA, USA). The reactions were amplified using the Rotor-Gene SYBR Green PCR Kit (Qiagen, Germany) according to the manufacturer’s protocol. The qRT-PCR was performed for 5 s at 95 °C, 30 s at 58 °C, and 10 s at 72 °C for 44 cycles and finalized by a melting curve analysis with 0.5 °C per 5 s from 65 °C to 95 °C (Table 3). The primers used to compare the expression of each gene are listed in Table 4.

### 3.4. Statistical Analysis

All experiments were repeated thrice. The callus induction rate was calculated as the number of explants with callus divided by the total number of explants and multiplied by 100. The data were verified for significance by analysis of variance (ANOVA) and Duncan’s multiple range test (DMRT) using the Statistical Analysis System (SAS) program.

## 4. Conclusions

We successfully induced callus from the petals of the rose breeding line 15R-12-2 by selecting the appropriate explant, explant’s position, and modified medium composition. The highest callus induction rate (65%) was observed when the petals of the FOF were placed on the SH11DP medium so that the abaxial surface was in contact with the medium. Thirty components, including hexanal, 6-methylhept-5-en-2-one, 2-ethylhexan-1-ol, (Z)-oct-2-en-1-ol, and nonanal were detected in the rose-petal-derived callus. In particular, the presence of a large amount of 2-ethylhexan-1-ol, the same VOC detected in the petals of 15R-12-2, was confirmed. In addition, hexanal (fresh grass scent) and 6-methylhept-5-en-2-one (tangerine-like lemongrass scent), which were not detected in petals, were also identified in the petal-derived callus. Callus containing these VOCs is expected to have a high value in the industry and can be proposed as an alternative pathway to obtaining volatile organic compounds.

## 5. Patent

M.S.A., S.J.K. and S.Y.L. are co-inventors on a patent (patent no. KR 10-2022-0159134) application related to the methods described in this manuscript.

## Figures and Tables

**Table 1:** Induction rates and morphological characteristics of callus under each culture condition.

Flower Stage	Orientation ^z^	Media	Induction Rate (%) ^y^		Color	Texture
Fully open	Abaxial	SH11D	30	ab	Light yellowish	Soft and Friable
		SH11DP	65	a	Light yellowish	Soft and Friable
		WPM11D	0	b	-	-
	Adaxial	SH11D	20	b	Light yellowish	Soft and Friable
		SH11DP	30	a	Light yellowish	Soft and Friable
		WPM11D	0	b	-	-
Partially open	Abaxial	SH11D	0	b	-	-
		SH11DP	40	ab	Light yellowish	Soft and Friable
		WPM11D	0	b	-	-
	Adaxial	SH11D	0	b	-	-
		SH11DP	40	ab	Light yellowish	Soft and Friable
		WPM11D	0	b	-	-

^z^ Means orientation of the petal disc attached to the medium. ^y^ Means followed by the same letter are not significantly different from each other at (*p* < 0.05) determined using Duncan’s multiple range test (DMRT).

**Table 2:** List of VOCs analyzed from the petal-derived callus of rose breeding line 15R-12-2 using GC-MS.

RI	CAS	Name	Formula	Molecular Weight (g/mol)	Relative Content (%)					Odor Description	Identification Method
					Hexane	Dichloromethane	Hexane: Dichloro-methane (1:5)	Methanol	Hexane: Methanol (1:5)		
758	2919-23-5	Cyclobutanol	C_4_H_8_O	72.11	-	-	-	0.08	-		MS
801	66-25-1	Hexanal	C_6_H_12_O	100.16	13.07	2.37	13.45	7.75	0.83	Fresh, green, fatty, aldehydic, grass, leafy, fruity, sweaty	MS, RI
833	553-90-2	Dimethyl oxalate	C_4_H_6_O_4_	118.09	-	-	-	22.83	-		MS
873	13952-84-6	Butan-2-amine	C_4_H_11_N	73.14	-	-	0.02	-	-	Fishy, ammonia	MS
953	7383-19-9	Hept-1-yn-3-ol	C_7_H_12_O	112.17	-	24.01	19.01	1.94	-		MS
986	110-93-0	6-methylhept-5-en-2-one	C_8_H_14_O	126.2	-	3.76	3.09	-	-	Citrus, green, musty, lemongrass, apple	MS, RI
991	3173-53-3	Isocyanatocyclohexane	C_7_H_11_NO	125.17	-	-	-	1.98	0.19		MS
998	124-18-5	Decane	C_10_H_22_	142.28	-	-	-	17.89	0.47		MS, RI
1011	17302-01-1	3-Ethyl-3-methylheptane	C_10_H_22_	142.28	-	-	-	-	1.83		MS
1027	104-76-7	2-ethylhexan-1-ol	C_8_H_18_O	130.229	59.01	9.74	5.28	1.80	0.62	Citrus, fresh, floral, oily, sweet	MS, RI
1042	821-97-6	(Z)-undec-3-ene	C_11_H_22_	154.29	-	-	-	-	0.05		MS
1058	1000309-20-1	2-ethylhexyl octadecyl sulfite	C_26_H_54_O_3_S	446.8	-	-	-	-	0.31		MS
1065	26001-58-1	(Z)-oct-2-en-1-ol	C_8_H_16_O	128.21	-	-	1.19	-	-	Sweet, floral	MS
1080	55170-80-4	2,4-dimethyldec-1-ene	C_12_H_24_	168.32	-	-	-	-	6.82		MS
1096	1000309-24-3	1-O-pentadecyl 2-O-prop-2-enyl oxalate	C_20_H_36_O_4_	340.5	-	-	-	0.27	-		MS
1099	3913-02-08	2-butyloctan-1-ol	C_12_H_26_O	186.33	-	-	-	0.33	-		MS
1101	124-19-6	Nonanal	C_9_H_18_O	142.24	-	-	-	0.30	-	waxy, aldehydic, rose, fresh, orris, orange, peel, fatty, peely	MS
1240	61141-72-8	4,6-dimethyldodecane	C_14_H_30_	198.39	-	-	-	0.62	-		MS
1276	31295-56-4	2,6,11-trimethyldodecane	C_15_H_32_	212.41	-	-	-	-	3.55		MS, RI
1301	91337-07-4	5-methyl-2-propan-2-ylheptan-1-ol	C_11_H_24_O	172.31	-	-	-	-	5.37		MS
1304	1000309-34-3	2-O-(6-ethyloctan-3-yl) 1-O-(4-methylpentyl) oxalate	C_18_H_34_O_4_	314.5	-	-	-	0.27	-		MS
1310	6750-34-1	3,7,11-trimethyldodecan-1-ol	C_15_H_32_O	228.41	-	-	-	2.53	-		MS
1315	13187-99-0	2-Bromo dodecane	C_12_H_25_Br	249.23	-	-	-	-	0.57		MS
1318	18675-24-6	2-methyldecan-1-ol	C_11_H_24_O	172.31	-	-	-	1.36	-		MS
1322	3891-98-3	2,6,10-trimethyldodecane	C_15_H_32_	212.41	-	-	-	0.73	-		MS
1366	1000406-39-2	1-heptoxydodecane	C_19_H_40_O	284.5	-	-	-	-	0.18		MS
1425	1009-61-6	1-(4-acetylphenyl)ethanone	C_10_H_10_O_2_	162.18	-	-	-	-	0.30		MS
1475	195194-80-0	1-(4-bromobutyl)piperidin-2-one	C_9_H_16_BrNO	234.13	-	-	-	-	0.08		MS
1492	504-44-9	Crocetane	C_20_H_42_	282.5	-	-	-	-	0.64		MS
1535	1000406-09-9	Tetradecyl €-hept-2-enoate	C_21_H_40_O_2_	324.5	-	-	-	-	0.08		MS

**Table 3:** qRT-PCR conditions for examining the relative expression levels of genes related to volatile organic compound biosynthesis between the petal and petal-derived callus of the rose breeding line 15R-12-2.

Stage		Temperature (°C)	Time	Cycles
Initial Denaturation		95	3 min	1
Amplification	Denaturation	95	5 s	44
	Annealing	58	30 s	
	Extension	72	10 s	
Melt Curve		65–95	0.5 °C increment 5 s/step	1

**Table 4:** Sequences of primers used to compare the expression of genes involved in volatile organic compound biosynthesis.

Gene Name	Primer Sequence (5′-3′)	
	Forward	Reverse
*OOMT1* ^Z^	CTGACCTGCAAGGAAGTAAGAA	TCGCTCCAGTCATGCAATATC
*OOMT2*	CTACCAATCCATCCAACCAAATC	GGGAAGCATCAGTAAGGGTATAA
*RhAAT1*	AGTTCACTCCCACAACGTATTT	AAAGTGAGAGCCTGGGAAAC
*RrNUDX1*	GGATGGTATGAGTGGGACAATC	GCTAGCAGCTGTCTCATGTT
*RhACTIN*	GTTCCCAGGAATCGCTGATA	ATCCTCCGATCCAAACACTG

^Z^*OOMT*: orcinol O-methyl transferase; *AAT*: alcohol acetyltransferase; *NUDX*: Nudix hydrolase.

## Data Availability

All data are contained within the article or Supplementary Materials.

## Supplementary Materials

The following supporting information can be downloaded at: https://www.mdpi.com/article/10.3390/plants12162986/s1, Figure S1: Component analysis of petals of the rose breeding line 15R-12-2 extracts by gas chromatography–mass spectrometry; Table S1: List of VOCs analyzed from petals of the rose breeding line 15R-12-2 using GC-MS.
