# Supplementary material for: Callus Derived from Petals of the Rosa hybrida Breeding Line 15R-12-2 as New Material Useful for Fragrance Production
Source: Plants (Basel). 2023 Aug 18;12(16):2986. doi: 10.3390/plants12162986 (PMC10457957; doi:10.3390/plants12162986)
Supplement: Supplementary file 1 [file plants-12-02986-s001.zip › plants-2532388-supplementary.pdf]

Supplementary data

Table S1. List of VOCs analyzed from petals of the rose breeding line 15R-12-2 using GC-MS.

RI	CAS	Name	Formula	Relative area (%)
2.063	56-41-7	Alanine	C3H7NO2	2.69
5.765	111-65-9	Octane	C8H18	0.04
7.893	106-72-9	5-Heptenal, 2,6-dimethyl-	C9H16O	0.94
8.539	42072-39-9	(S)-(+)-3-Methyl-1-pentanol	C6H14O	10.17
14.613	123-35-3	beta-myrcene	C10H16	1.52
15.132	99-83-2	alpha-phellandrene	C10H16	0.1
15.413	3681-71-8	3-Hexen-1-ol, acetate, (Z)-	C8H14O2	7.34
15.821	142-92-7	Acetic acid, hexyl ester	C8H16O2	18.18
15.948	56922-75-9	2-Hexen-1-ol, acetate, (Z)-	C8H14O2	3.68
16.165	527-84-4	o-Cymene	C10H14	0.08
16.34	5989-27-5	D-Limonene	C10H16	0.66
16.525	104-76-7	2-Ethyl-1-hexanol	C8H18O	5.11
16.98	7785-70-8	1R-(+)-α-Pinene	C10H16	0.23
17.266	816-19-3	Methyl 2-ethylhexanoate	C9H18O2	0
17.454	13877-91-3	beta-ocimene	C10H16	1.22
17.891	99-85-4	gamma-terpinene	C10H16	0.06
19.368	586-62-9	terpinolene	C10H16	0.17
19.919	15186-51-3	rosefuran	C10H14O	0.23
20.073	539-52-6	Perillene	C10H14O	1.31
20.592	1960-12-08	Phenylethyl Alcohol (2PE)	C8H10O	6.58
21.095	36262-09-6	2,4-thujadiene	C10H14	0.04
21.476	460-01-5	Cosmene	C10H14	0.24
22.053	7216-56-0	(E,Z)-alloocimene	C10H16	0.05
22.636	103-09-3	2-ethyl hexyl acetate	C10H20O2	0.24
24.357	98-55-5	alpha-terpineol	C10H18O	0.02
24.764	140-67-0	Estragole	C10H12O	0.01
24.849	1000131-87-6	2-Methyl-3-phenyl-propanal	C10H12O	0.02
25.786	13066-51-8	gamma-isogeraniol	C10H18O	0.01
25.919	629-06-1	1-Chloroheptane	C7H15Cl	0
26.57	41436-42-4	delta-damascone	C13H20O	0.06
26.777	106-26-3	Neral	C10H16O	0.14
26.957	5944-20-7	Isogeraniol	C10H18O	0.01
28.026	4179-19-5	3,5-Dimethoxytoluene (DMT)	C9H12O2	17.73

28.164	5392-40-5	Citral	C10H16O	0.35
28.323	872858-42-9	4,8-Dimethylnona-3,8-dien-2-one	C11H18O	0.07
28.646	1862-61-9	Methyl nerate	C11H18O2	0.03
30.468	3209-13-0	3-Methoxy-5-methylphenol	C8H10O2	0.01
30.589	1189-09-9	(E)-methyl geranate	C11H18O2	3.25
31.204	1000159-45-7	Bicyclo[2.2.1]heptane, 2-cyclopropylidene-1,7,7-trimethyl-	C13H20	0.01
31.881	150-84-5	Citronellyl acetate	C12H22O2	0.24
32.109	51468-85-0	Megastigma-4,6(Z),8(E)-triene	C13H20	0.06
32.692	3856-25-5	Copaene	C15H24	0.01
33.73	32659-21-5	Ethyl geranate	C12H20O2	0.07
33.857	629-59-4	Tetradecane	C14H30	0.04
34.047	93-15-2	Methyleugenol	C11H14O2	0.19
34.153	621-23-8	1,3,5-Trimethoxybenzene (TMB)	C9H12O3	0.02
34.508	118-65-0	Isocaryophyllene	C15H24	0.4
35.376	17283-81-7	Dihydro-beta-ionone	C13H22O	0.06
35.662	3293-47-8	Dihydro-beta-ionol	C13H24O	0.24
35.917	1000062-61-9	1,4,7,-Cycloundecatriene, 1,5,9,9-tetramethyl-,Z,Z,Z-	C15H24	0.01
36.033	3796-70-1	Dihydro-beta-ionol	C13H22O	0.01
36.917	30021-74-0	gamma-muurolene	C15H24	0.01
37.055	1000365-95-5	cis-muurola-4(14),5-diene	C15H24	0.01
37.315	79-77-6	trans-beta-Ionone	C13H20O	0.04
37.881	31983-22-9	alpha-muurolene	C15H24	0.05
37.95	629-62-9	Pentadecane	C15H32	0.11
45.517	629-78-7	Heptadecane	C17H36	0.17
51.474	31035-07-1	9-Nonadecene	C19H38	0.22
52.348	629-92-5	Nonadecane	C19H40	0.63

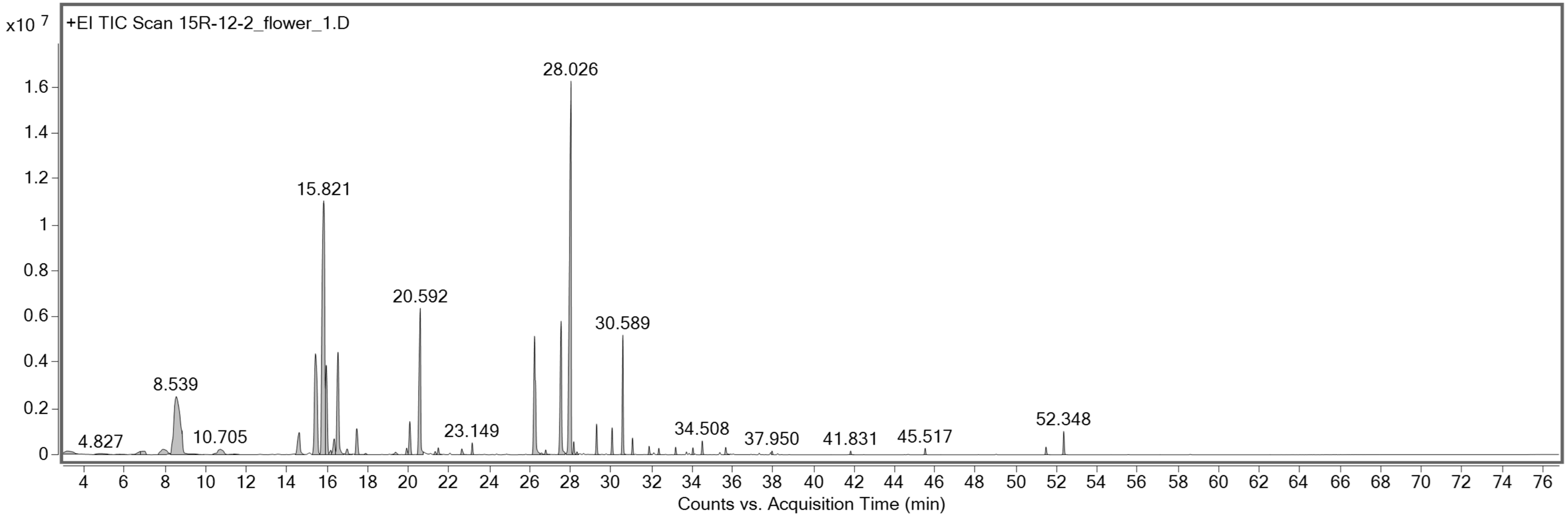

Figure S1. Component analysis of petal extracts of the rose breeding line 15R-12-2 by gas chromatography–mass spectrometry.
